# An evaluation of HHV-6 as an etiologic agent in Hodgkin lymphoma and brain cancer using IARC criteria for oncogenicity

**DOI:** 10.1186/s13027-019-0248-3

**Published:** 2019-11-05

**Authors:** Michael J. Wells, Steven Jacobson, Paul H. Levine

**Affiliations:** 1 School of Community and Population Health, University of New England, 716 Stevens Ave, Portland, ME 04103 USA; 2 National Institutes of Health, National Institute of Neurological Disorders and Stroke, Viral Immunology Section, 9000 Rockville Pike, Bethesda, MD 20892 USA; 3 College of Public Health, University of Nebraska, 984355 Medical Center, Omaha, NE 68198 USA

**Keywords:** HHV-6, Human oncogenic virus, Hodgkin’s lymphoma, Hodgkin lymphoma, Hodgkin’s disease, Brain cancer, Oral cancer

## Abstract

**Background:**

Human herpesvirus-6 (HHV-6) is a ubiquitous double-stranded DNA virus that can cause roseola infantum, encephalitis, and seizure disorders. Several studies have shown an association between HHV-6 and cancer but confirmation of an etiologic role is lacking. We reviewed the criteria for viral causation of cancer used by The International Agency for Research on Cancer (IARC) for six oncogenic viruses and applied criteria to published reports of HHV-6 and its association with Hodgkin lymphoma and brain tumors.

**Methods:**

Our major criteria for oncogenicity were finding evidence of the virus in every tumor cell and prevention of the tumor by an antiviral vaccine. Our six minor criteria included: 1) suggestive serologic correlation, such as higher virus antibody levels in cases compared to controls; 2) evidence of the virus in some but not all tumor cells, and 3) time space clustering. We focused on Epstein-Barr virus (EBV) as the primary virus for comparison as HHV-6 and EBV are both Herpesviridae, ubiquitous infections, and EBV is well-accepted as a human oncovirus. Particular attention was given to Hodgkin lymphoma (HL) and brain cancer as these malignancies have been the most studied.

**Results:**

No studies reported HHV-6 satisfying either of the major criteria for oncogenicity. Of the minor criteria used by IARC, serologic studies have been paramount in supporting EBV as an oncogenic agent in all EBV-associated tumors, but not for HHV-6 in HL or brain cancer. Clustering of cases was suggestive for both HL and brain cancer and medical intervention suggested by longer survival in patients treated with antiviral agents was reported for brain cancer.

**Conclusion:**

There is insufficient evidence to indicate HHV-6 is an etiologic agent with respect to HL and brain cancers. We suggest that methods demonstrating EBV oncogenicity be applied to HHV-6. It is important that one study has found HHV-6 in all cancer cells in oral cancer in a region with elevated HHV-6 antibodies and therefore HHV-6 can still be considered a possible human oncogenic virus.

## Background

Human herpesvirus-6 (HHV-6) is a ubiquitous infectious agent that is the primary cause of roseola infantum, an acute childhood illness characteristically presenting with a high fever followed by a generalized rash. Other manifestations of primary infection with HHV-6 have been investigated [1] and current research is focusing on its neurotropic properties suggesting a link to encephalitis, seizure disorders, Alzheimer’s disease [2], and perhaps multiple sclerosis [3, 4]. The association of HHV-6 with cancer dates back to the first report of its isolation from patients with hematologic malignancies. Initially described as a human B-cell lymphotropic virus (HBLV) [5], HHV-6 has now been shown to be more pleotropic, growing well in CD4+ T lymphocytes [6–8] and also able to replicate in macrophages and fibroblasts among other cells [1].

Initially, two strains of HHV-6, designated as HHV-6A and HHV-6B, were defined with differences noted in geographic distribution, primary reservoir areas, and associated diseases in humans [1, 3, 9]. More recently, these two agents have been classified as distinct viruses [9]. Both are closely related *Herpesviridiae* in the beta subfamily of *Herpesviridiae*, along with cytomegalovirus and HHV-7. In contrast, Epstein-Barr virus (EBV), another member of the *Herpesviridiae* family, is classified in the gamma herpesvirus subfamily. The two HHV-6 viruses have a unique form of latency. Unlike EBV, they do not form episomes but rather establish latency by integrating near the telomere of the chromosome [10].

HHV-6B appears to be spread primarily through saliva [3], although it has been detected in stool samples [11, 12] and vaginal secretions [13]. It is commonly transmitted from mother-to-infant. HHV-6A is more prevalent in adults compared to children, and to date has not been definitively associated with human disease, unlike HHV-6B [1]. The pattern of spread of different infectious oncogenic agents has been important in indicating the relationship to human cancer. Human T-cell lymphotropic Virus Type-I (HTLV-I), for example, is highly cell-associated and not readily transmissible. There is a high prevalence of this virus in only a few areas, particularly Japan and the Caribbean [14–16]. Therefore, the strong geographic correlation between the diseases resulting from infection with this virus, such as adult T-cell leukemia/lymphoma (ATLL) and HTLV-I associated myelopathy (HAM), constitutes strong support for the etiological role of HTLV-I in those diseases.

As molecular techniques have advanced, the criteria for determining whether an infectious agent causes cancer have changed. The classic criteria of disease causation is long-standing and includes suggestions from Henle and Koch [17–19], Bradford Hill [20], Rivers [21], and Fredericks and Relman [22], who focused on detection of the virus by in situ methods in each of the tumor cells. Under this direct hit model, the agent transforms an initially healthy cell into a malignant cell and thereafter persists in all of the subsequent tumor cells. Moore and Chang [23] recently used newly developed molecular techniques to implicate HHV-8, a gamma herpesvirus, as the cause of Kaposi’s sarcoma, and the Merkel Cell tumor virus (MCV), a polyomavirus, as the cause of Merkel cell carcinomas. However, with all of these agents, it is unclear if the continued presence of the virus is required to maintain the tumor once oncogenesis is initiated.

Other mechanisms of oncogenesis have been described, primarily through chronic inflammation which causes cellular proliferation. Hepatitis C virus (HCV) causes hepatocellular carcinoma through the intermediary of hepatic cirrhosis; non-viral infectious agents also cause cancer via chronic inflammation (e.g. *Helicobacter pylori* and gastric cancer, *Schistosom*a *haematobium* and bladder cancer, and *Opisthorchis viverrini* and bile duct cancer) [24].

As we have learned more about human oncogenic agents, it is clear that some of the early criteria for disease causation, such as specificity (a one-to-one relationship or singular causal agent causing a singular disease posed by Bradford Hill), do not apply to oncogenic viruses. Gastric cancer appears to have at least two infectious etiologic triggers, *H. pylori* [25, 26] and Epstein-Barr virus [27–29]. Hepatocellular cancer can be caused by HCV or Hepatitis B virus (HBV) through entirely different mechanisms and can also occur with any chronic hepato-destructive process. Epstein-Barr virus is etiologically linked to several tumors: Hodgkin lymphoma (HL) in approximately 36% of cases [30], Burkitt lymphoma in the vast majority of cases in sub-Saharan Africa, non-keratinizing nasopharyngeal carcinoma (NPC) in almost every case worldwide, as well as other malignancies [27–29, 31, 32].

Currently, the International Agency for Research on Cancer (IARC) develops consensus panels and rates the oncogenic potential in three groups: Group 1—carcinogenic for humans; Group 2—sufficient data to conclude either probably carcinogenic for humans (2A) or possibly carcinogenic for humans (2B); and Group 3—not classifiable as carcinogenic for humans, which can mean either no evidence favoring a link or no studies examining the issue of carcinogenicity. We reviewed the IARC criteria for the six viruses that have been evaluated in IARC monographs for their criteria for oncogenicity and applied these criteria to published reports of HHV-6 and its association with Hodgkin lymphoma and brain tumors.

## Methods

First, we listed the most important criteria for oncogenicity identified by IARC for the Group 1 viral carcinogens. The six viruses classified as Group 1 carcinogens were reviewed: Epstein-Barr virus (EBV) [33], Human Herpesvirus 8 or Kaposi’s sarcoma herpesvirus (HHV-8, KSHV) [34], Human T-lymphotropic virus Type I (HTLV-I) [35], Hepatitis B and C viruses (HBV and HCV) [33, 36, 37], and human papilloma virus (HPV) [33, 38, 39]. We did not include MCV as it has not been classified in Group 1 at this time, the latest review stating “There is *limited evidence* in humans for carcinogenicity” [40]. Also, we did not include the human immunodeficiency virus (HIV) because it is not directly oncogenic but rather causes cancer through the associated immunodeficiency.

We have chosen to divide the criteria for etiology into two groups: major criteria (those that generally by themselves are accepted as proving etiology and are invariably accompanied by a number of minor criteria) and minor criteria, (those that are frequently found in oncogenic viruses certified as Group 1 carcinogens (see below) by the IARC). For major criteria, we have chosen two findings that are generally accepted by viral oncologists as providing the strongest evidence for oncogenicity: viral evidence in all tumor cells and tumor prevention by an antiviral vaccine.

### Major criteria

#### Virus detection studies

Strong evidence of causality should begin with viral evidence in all tumor cells, which we designate as “diffuse” involvement to distinguish this from viral evidence in some tumor cells, which we designate as “restricted” involvement. Clearly, many cells can be infected by viruses without consequence, but this model assumes that malignant transformation initially occurs in one cell, and then this transformed cell carries viral information to its progeny. Because of this carriage, presence of the virus is constantly maintained in tumor cells. Examples of this criterion include EBV and undifferentiated nasopharyngeal carcinoma [41], EBV-associated gastric cancer [42–44], and EBV-associated Hodgkin lymphoma [45–48]. As a necessity for this to be accepted as proof of causation, we added the criterion of reproducibility with confirmation of persistent carriage established by independent laboratories.

#### Tumor prevention

Another major criterion that is generally accepted and noted by Evans and Mueller [49] is prevention of the tumor by blocking infection with the suspected etiologic agent. Thus far, only the HBV vaccine has met this criterion [50] although the HPV vaccine has effectively reduced the number of pre-cancerous cervical lesions and will likely reduce the incidence of cervical cancer in the immunized populations [51].

### Minor criteria

Minor criteria are important clues to oncogenicity and are often found to exist together in the same candidate agent. The criteria include causative evidence in serologic and viral detection studies, biological gradient, clustering, medical interventions, and animal models.

#### Serologic studies

Serologic studies usually provide the first evidence for an association of an oncogenic virus and a disease. Numerous examples exist which have shown higher antibody titers in the associated disease than controls for all of the accepted oncogenic viruses. However, such studies can provide false leads, such as the initial focus on herpes simplex virus 2 (HSV-2) as the cause of cervical cancer [52]; both HSV-2 and the oncogenic strains of human papillomavirus are sexually transmitted.

#### Case-control studies

Case-control studies provide some refinement since the cases are specifically matched by age, sex, and other relevant criteria, but as with HSV-2, if the confounding agent is transmitted in the same way as the etiologic agent (as in this case HPV) careful matching is of little help. Examples of useful case-control studies cited by IARC include reports on African Burkitt lymphoma (BL) patients by Henle et al. [53] and Klein et al. [54]. However, higher antibody level might also occur when a passenger virus infecting the tumor replicates with the tumor even if it infects the tumor after it has started. Furthermore, tumor-induced cachexia might affect immune responses to a virus.

#### Cohort studies

Cohort studies provide stronger evidence than case-control studies, being able to determine if subjects who developed disease had higher antibody titers in the pre-disease sera. For EBV, a large prospective study [55] funded by the National Cancer Institute showed that high EBV antibody predicted the development of BL. Second, the BL patients whose EBV antibody titers were lower than the general population and whose tumors could be studied had non-EBV associated BL, i.e., BL without the virus being present in the tumor cells. Similarly, Beasley et al. showed that Taiwanese subjects infected with HBV had a 223-fold higher risk of developing hepatocellular carcinoma than uninfected controls [56]. This prospective study resulted in a HBV vaccine trial which eventually demonstrated that preventing infection led to a decrease in mortality from the associated cancer [50], thus meeting one of our major criteria for causality.

#### Retrospective-prospective cohort studies

Retrospective-prospective cohort studies utilize already collected serum samples and select sera to test based on the outcome. For example, based on Shibata’s description of EBV in a series of gastric carcinomas [42], we selected sera from the Honolulu Heart Program cohort study of 8006 American men of Japanese ancestry and tested sera from 54 cases of non-lymphoepithelial gastric carcinoma collected between 2.7 and 21.3 years (median 14 years) prior to the diagnosis of cancer. This study, which essentially allowed the same approach as the prospectively collected sera from Ugandan children [55], showed higher antibody titers to EBV in the pre-EBV positive gastric cancer patients compared to controls, whereas mean Helicobacter pylori antibody reactivity was higher in the sera prior to development of EBV-negative gastric cancer patients and controls [57]. Coincidentally, antibody to HHV-6 was also tested in this study and no difference between groups was noted.

##### Virus detection studies

As noted above, viral evidence in every cell (diffuse involvement) and none in non-malignant cells is convincing evidence of viral etiology, and is considered a major criterion. However, virus detection in only some of the tumor cells (restricted involvement) is common. Tumors may contain infiltrating cells which can introduce the viruses they carry. Or they may be heterogeneous (e.g., Hodgkin lymphoma is a mixture of tumor and reactive cells). Thus, detection does not necessarily signal etiology and all cells cannot be expected to carry the virus, even if that virus is etiologic. Moreover, some antibody reagents only detect the virus in certain phases of replication in the malignant cell (e.g., early EBV studies and cancer where EBV viral capsid, membrane and other antigens were found inconsistently). Uniform detection in all tumor cells requires the identification of EBV-encoded RNAs or (EBERs), which are expressed in all forms of EBV latency [45–48]. Thus, virus detection with restricted involvement is considered a minor criterion.

##### Biological gradient

Both serologic and virus detection studies can provide important information as to biologic gradients. Overall, these studies analyze the relationship between viral load and susceptibility to disease and/or severity of disease. There are numerous examples of viral load paralleling disease burden, which can be reflected in antibody levels. Correlation of antibody titers or viral load with the presence and particularly the stage of disease can be helpful, as in BL and NPC where antibody titers to EBV-associated early antigen clearly parallel disease burden [58, 59]. Similarly, virus burden may signal higher tumor risk. With HTLV-I, virus load or elevated viral antibodies in the mother indicate the likelihood of the virus being passed on to the infant at birth or in the neonatal period, which is the primary risk factor for the development of ATLL [60].

##### Clustering

These studies are particularly useful where the virus is not readily transmitted, such as with HTLV-I which is highly cell-associated and is transmitted by breast feeding, transfusion of blood cells or sexually. The finding that the HTLV-I endemic areas were precisely the same as the ATLL endemic areas [14, 61] was the first convincing epidemiologic link of the virus with the malignancy. A similar finding is seen with classic Kaposi’s sarcoma, which was shown to concentrate in southern Italy which has a higher prevalence of HHV-8 antibodies than northern Italy [62]. This criterion has not been used for HHV-6 since it is a ubiquitous virus worldwide but geographic differences in titers in different populations have been noted [63].

##### Medical interventions

If it is demonstrated that antiviral treatment results in decreasing the virus burden resulting in reduced pathogenesis and subsequent cancer, this could be a clue that the virus load is important in cancer etiology. Currently, this approach is promising for antiviral treatment of HCV and HBV to reduce the incidence of hepatocellular carcinoma. Reduction of the burdens of bacterial *H. pylori* and parasites such as *O. viverrini* are being employed to reduce the incidence of gastric cancer and bile duct cancer respectively in endemic areas.

##### Animal models

In some instances, oncogenicity is demonstrated by inoculating animals with the human virus and producing a similar tumor, as suggested by the production of leukemic tumors in immune-defective mice inoculated with HTLV-1-containing cells [64]. More often, a naturally occurring animal tumor is found to be produced by a virus that resembles the human tumor. Early studies investigating the oncogenicity of EBV for humans relied on observations of Marek’s disease, a lymphoma of chickens produced by the Marek’ disease herpes virus which closely resembles EBV [65]. Also, naturally occurring feline leukemia virus produces diseases in cats that have been considered a useful model for HTLV-I-associated ATLL in humans [66].

With these major and minor criteria in mind, we evaluated the above criteria in the two human malignancies that have had the most data suggesting an etiologic role for HHV-6, the nodular sclerosis form of Hodgkin lymphoma and brain cancer. We attempted to identify all peer-reviewed manuscripts published on the association of HHV-6 with Hodgkin lymphoma or brain cancer. A systematic search was performed on PubMed using combinations of the search terms “human herpesvirus 6, human herpesvirus-6, HHV6, HHV-6” and “Hodgkin’s lymphoma, Hodgkin lymphoma, Hodgkin’s disease, Hodgkin disease,” or “brain cancer, brain tumor, brain tumors, glioma,” for a total of 36 separate searches. In addition, several review articles on HHV-6 and Hodgkin lymphoma or brain cancer [67–69] were reviewed for identification of relevant manuscripts that may not have been identified in our search.

## Results

Thus far, no human tumor has been linked to HHV-6 by either of our major criteria. The status of the minor criteria for Hodgkin lymphoma and brain cancer are described below and summarized in Table 1.

**Table 1:** IARC criteria for oncogenicity for six oncoviruses with HHV-6 comparison

Criteria	Characteristics	EBV	HTLV-1	HHV-8	HPV	HBV	HCV	HHV-6
Major criteria	Viral detection (diffuse)	X	X	X	X	X		
	Tumor prevention (by antiviral vaccine)				^a^+/−	X		
Minor criteria	Serology	X	X	X	X	X	X	
	Viral detection (restricted)	N/A	N/A	N/A	N/A	N/A		X
	Biological gradients	X	X	X		X	X	
	Clustering		X	X		X	X	^b^+/−
	Medical interventions				X	X	X	X
	Animal models	X	X			X		

^a^Vaccine reduces number of precancerous lesions [51]

^b^Some evidence supports clustering of HHV-6 [70, 71]

### Hodgkin lymphoma

Seventy-three peer-reviewed publications evaluating HHV-6 in Hodgkin lymphoma were reviewed for the criteria listed above. Eight studies with serologic data were identified [69, 72–78].

#### Serologic studies

The initial case-control serologic studies evaluating antibodies to HHV-6 in Hodgkin lymphoma were based on samples of convenience and generally showed higher antibody titers in patients with HL compared to population controls. Ablashi et al. [72] used an immunofluorescence assay beginning at a 1:20 dilution, finding only 26% of healthy donors in the U.S. Canada and Europe were positive compared to 77.2% of patients with Hodgkin lymphoma [72]. Clark et al. examined sera from patients with diverse hematologic malignancies which included two groups of HD patients, using various dilutions of antibody detected by immunofluorescence [73]. They observed elevated titers in HL but also in acute myeloid leukemia and low grade non-Hodgkin lymphoma [73]. Other investigators, including Biberfeld et al. and Torelli et al., also found higher antibody titers in HL than in various control groups [74, 75].

In contrast, Levine et al. obtained divergent results in 37 longitudinally-followed patients with HL in Denmark tested for HHV-6 by both immunofluorescence assay (IFA) and enzyme-linked immunosorbent assay (ELISA) [76]. The major findings in this study were: 1) Initial pre-treatment samples showed lower HHV-6 antibody titers in HL patients compared to controls. 2) ELISA and IFA results were discordant, with IFA rising and ELISA declining over time showing the importance of the assay used. 3) Antibodies showed major changes with treatment in HL cases but were remarkably stable in healthy controls studied longitudinally. 4) Both EBV and HHV-6 IFA titers rose longitudinally in relapsed but not in patients who did not relapse. No studies other than, Levine, et al. [76], have examined HHV-6 antibodies using prospective cohort or retrospective-prospective cohorts, although such approaches provided important etiological data for EBV.

#### Virus detection studies

The initial virus detection studies [75, 77, 78] looked for the presence of HHV-6 in HL tissues, but only one determined the specific location of the virus in Reed-Sternberg (RS) cells, the tumor cell in HL [77]. HHV-6 was not only found in HL but also in other lymphomas and in non-malignant reactive lymph nodes [79]. One study by Torelli et al. [74] exemplified the pattern in several reports suggesting that while the percentage of HL cases with HHV-6 detected in the lymph nodes was small, the possibility remained that a subset of cases such as three young women with similar clinical and histologic subtypes of HL reported as nodular sclerosis-lymphocyte depletion HL could be etiologically linked to HHV-6.

As noted above, one major criterion for viral oncogenicity is finding evidence of the virus in every tumor cell, suggesting the virus was the precipitating oncogene carried along with every subsequent mitotic division. In her review of viruses possibly related etiologically to HL, Jarrett [69] summarized the HHV-6 virus detection studies and noted that no report to date has documented this finding. A relevant paper of interest by Luppi et al. [80] looked for HHV-6 expression in a variety of benign and malignant diseases, finding viral late antigen expression in sinus histiocytosis with massive lymphadenopathy (SHML or Rosai-Dorfman disease) confirming our original report in 1992 [81]. However, Luppi et al. found HHV-6 was detected rarely in HL, and when it was identified in Reed-Sternberg cells, the cells were degenerating indicating a lytic process, not compatible with the continued perpetuation of the virus in constantly dividing malignant cells. Another intriguing paper by Siddon et al. [77] reported using immunohistochemistry, polymerase chain reaction (PCR), and fluorescent in situ hybridization (FISH) to localize the virus in Hodgkin tissues. Lymph nodes from 21 HL patients, nodular sclerosis subtype, were selected and 18 (86%) were HHV-6 positive with 10 of these containing HHV-6-positive RS cells. Four different monoclonal antibodies (to whole virus, HHV-6 p41 late antigen, p98 late antigen, and U94 latent antigen), as well as FISH, identified the virus in the RS cells. The author’s note that the identification of multiple copies of the virus detected by FISH is suggestive of viral replication in some RS cells, and photomicrographs document “numerous HHV-6 positive Reed-Sternberg (RS) cells” but the percentage of positive RS cells was not recorded.

#### Biological gradient

HHV-6 DNA has been identified in white blood cells and plasma of pediatric HL patients [82], suggesting the opportunity for viral load studies. Thus far, however, we have not identified any studies that evaluate biologic gradient (correlation of viral load with the presence and particularly the stage of disease or DNA copy number in PCR detection studies of tumors).

#### Clustering

Time-space clustering, an intriguing observation suggesting an acute triggering event, is more likely to be revealing with aggressive malignancies with a rapid doubling time, such as acute leukemia [83], Burkitt lymphoma [84, 85] or inflammatory breast cancer [86, 87]. HL is far less aggressive but other approaches to clustering and transmissibility of an infectious agent have been reported and analyzed [70, 88]. The initial reports were particularly focused on high school students and the nodular sclerosis form of this lymphoma. The transmission of the proposed etiologic agent has been suggested as either being directly from patient to patient or via a single clinically unaffected “carrier” and the median interval between the diagnosis of the infective patient or clinically unaffected carrier and the infected patient has been estimated as 3 years [70, 88].

#### Medical interventions

No medical intervention studies were identified.

#### Animal studies

No animal studies were identified.

### Brain Cancer

Eleven studies were found for brain cancer [67, 89–98].

#### Serologic studies

One report by Cuomo et al. [95] showed no difference in the frequency of sera positive for HHV-6 in 73 patients with brain tumors compared to 150 healthy subjects. Only patients with brain tumors, however, had antibody titers > 1:320, suggesting that there could be a small subset of patients with brain tumors triggered by HHV-6 or reacting anomalously to it.

#### Virus detection studies

HHV-6 has been shown to be neurotropic and pathogenicity for humans has been well documented [67]. Since HHV-6 had demonstrated tropism for normal and neoplastic cells of neuronal and glial origin [97], it was reasonable to investigate the presence of HHV-6 DNA sequences by PCR in brain tumor specimens. Early work on the association of HHV-6 with brain cancer was studied by older, less sophisticated PCR techniques. Luppi et al. used frozen and paraffin-embedded samples from different regions of brains obtained at necropsy from 9 control immunocompetent adults and 37 cases of primary brain tumors of neuroglial origin [97]. Six normal brain tissues (66%) were positive for HHV-6 whereas only 7 of 37 (19%) brain tumors were positive. Similarly, Chan et al. [96] used a nested HHV-6 PCR method from 110 formalin fixed, paraffin-embedded surgical biopsies of primary brain tumors and found only 8.2% were positive (no non brain-tumor samples were analyzed).

By contrast, Crawford and colleagues had a series of studies noting HHV-6A and 6B in brain tumors of children and adults that focused on the possible role of these viruses in pediatric and adult gliomas [89, 90]. These reports used an HHV-6 nested PCR methodology, immunohistochemistry, and in situ hybridization studies. HHV-6 was more often detected in the tumor samples compared to age-matched non-tumor brains [89]. Of interest is the observation that the HHV-6A variant was more often detected. The authors appropriately note that these studies are more associative than causal, and do not suggest that HHV-6 is a direct cause of brain tumors. These early results were later supported by Chi, et al., [92] that investigated the frequency of herpesviruses in 40 glioma tissues compared to 13 normal brains by using both immunohistochemical (IHC) analysis and nested PCR. Of the 5 human herpesviruses tested (HSV-1, EBV, Cytomegalovirus (CMV), HHV-6 and HHV-7), only HHV-6 was detected significantly more frequently in gliomas (42.5%) than in normal brain (7.7%). Moreover, these observations were further supported by positive IHC staining for HHV-6 in a subset of the tumor samples (32.5%) and never observed in normal brain tissue [92]. Of all the different types of glioma tissues, 50% of glioblastoma multiforme (GBM) specimens were HHV-6 positive.

Other studies on GBM have used a novel and highly precise multiplexed droplet digital PCR to assay from 112 brain tissue specimens that included 45 GBM and 49 control brains [98]. Each tissue section was analyzed for HHV-6A, HHV-6B, EBV and CMV. Neither CMV nor HHV-6A were detected in any of the brain samples, while HHV-6B and EBV had a higher frequency of detection in the GBM samples compared to controls [98]. This cross-sectional survey of multiple human herpesviruses in high and low grade astrocytomas, as with subsequent studies, only showed that multiple herpes viruses can be detected in brain tumors.

#### Biological gradient

No studies were identified.

#### Clustering

As with HL, the latent period between the initiation of a brain tumor and its clinical appearance is far longer than with those aggressive malignancies reported to having a closer pattern of time-space clustering to an infectious disease. Evidence for this long latent period is provided by studies of known oncogenic agents, such as radiation, and the onset of the brain tumor, as observed by the experience in Israel where radiation for tinea capitis was shown to cause subsequent brain cancer many years later [99, 100]. One observation, possibly relevant to HHV-6, was a transient increase in brain cancer following a cluster of chronic fatigue syndrome (CFS) [101, 102] that was linked to HHV-6 [71].

CFS clusters, originally designated epidemic neuromyasthenia [103], have been reported since 1959 [104] and can apparently be triggered by different etiologic agents [105–108]. We focused on this particular outbreak in the Lake Tahoe region [109] affecting northern Nevada and nearby California because the reporting physicians believed they were now seeing a surge of cancer patients in their practice. Preliminary studies were conducted using the Nevada Cancer Registry and two malignancies, non-Hodgkin lymphoma (NHL) and brain cancer. These were all identified as occurring shortly after the CFS cluster in northern Nevada and were not seen in southern Nevada [106, 107]. The link between CFS and NHL was confirmed in a large study of CFS and cancer. This involved more than a million individuals greater than 65 years of age, combining two large data bases from the Surveillance, Epidemiology and End Results (SEER) program and Medicare. NHL was the only cancer with a statistically significant link to CFS [110]. A subsequent review of an additional 10 years of data from the Nevada Cancer Registry [111] found that the increase in brain cancer in northern Nevada remained statistically significant but was restricted to only those under age 65 and was therefore were not observable in the NCI SEER-Medicare analysis.

#### Medical interventions

There are few interventional clinical trials that have targeted HHV-6-associated cancers. However, there was one controversial report that described 50 patients with GBM who received valganciclovir as an add-on to standard therapy [112]. In the valganciclovir/GBM study, there was a higher rate of 2-year survival (62%) in GBM patients treated with this anti-beta herpesvirus drug than contemporary controls (18%) not receiving valganciclovir [112]. However, selection bias may have influenced these results [113], and therefore antiviral treatment in GBM should be extended to a properly designed randomized trial.

#### Animal studies

A recent study on the role of HHV-6 in chronic progressive neurologic disease may help to inform our understanding on how these viruses may act to trigger or accelerate disease [114]. Recently, it has been reported that HHV-6 infection in marmosets, a non-human primate, by intranasal route [115] resulted in a benign, quiescent infection in which viral material was recovered from peripheral compartments. Subsequent challenge of these animals with CNS antigens resulted in an autoimmune encephalomyelitis (EAE), which was more severe in animals that had been infected with HHV-6 than in uninfected animals in which EAE was induced [114]. Mechanistically, expansion of proinflammatory CD8+ T cells correlated with post-EAE survival in the virus inoculated animals suggesting that a peripheral antigen-driven expansion may have occurred [114]. These studies suggested that viral infection with a ubiquitous virus such as HHV-6 can ‘prime’ disease development in challenge models in which the challenge alone rarely induces disease [116]. Moreover, peripheral CNS viral infections can result in an enhanced response to subsequent autoimmune challenge and supports the hypothesis that viruses such as HHV-6 may act as triggers for inflammatory mediated neurologic disease or even CNS tumors.

## Discussion

The criteria for causation have changed over the years. We have evaluated the available data on HHV-6 as a human carcinogen using the criteria for accepted viral carcinogens such as EBV, HPV and HTLV-I by current standards and acceptance by IARC as a Group 1 carcinogens. For all of the viral carcinogens, EBV appears to be the best model for HHV-6 as it is ubiquitous worldwide and both are herpesviruses.

For our major criteria, only identification of the virus in all of the tumor cells is relevant since HHV-6 infection is not considered preventable. Attempts to apply an EBV vaccine to prevent specific tumors in high risk areas, such as NPC in southern China, have been considered [117] but not yet applied. Thus far, HHV-6 has not been demonstrated in every tumor cell in either HL or brain cancer. For Hodgkin lymphoma, EBV has been accepted as the cause of up to 30% of cases by identification of EBERs in every Reed-Sternberg cell [45, 46, 49, 69]. HHV-6 is also sometimes found in Reed-Sternberg cells but only in occasional cells (restricted). HHV-6 has been reported as present in all tumor cells (diffuse) in oral cancer, thus meeting this major criterion [118], but this single report remains unconfirmed.

In regard to minor criteria, there are possible opportunities to examine whether HHV-6 meets any of the criteria used to show EBV oncogenicity. Linking a human virus to cancer usually begins with serology. As described in an extensive review [119], elevated HHV-6 titers have been observed in patients with numerous human tumors. For EBV, however, the titers have universally been elevated in the sera of individuals destined to get the tumors years to decades in advance of diagnosis. In addition to Burkitt’s lymphoma [55] and gastric cancer [57], nasopharyngeal carcinoma is now an important malignancy where EBV serology has proven effective in screening of very high risk populations, such as high risk families [120]. Further studies will improve the specificity of assays that had earlier been used for larger populations [76].

As we have noted, all of the reports showing high HHV-6 titers have been done in treated patients. To our knowledge, the only study using sera from untreated HL patients showed no elevation compared to controls (and indeed were somewhat lower than controls); in that study, titers increased only after treatment [76]*.* Similar prospective longitudinal studies should be developed with HHV-6, so that findings parallel to EBV findings can be examined. It would be of value to use some of the large serum banks such as the prostate, lung, colorectal, and ovarian cancer screening trial (PLCO) where large prospective studies collect blood and other biological samples on clinically healthy individuals years before tumors were diagnosed to determine risk factors for subsequent disease, including cancer.

Most of the other minor criteria such as viral load pre-disease, medical intervention and animal models have not demonstrated HHV-6 to be related to either Hodgkin lymphoma or brain cancer with the exception of the valgancyclovir study in glial tumors (not yet confirmed). For both Hodgkin lymphoma and brain cancer, there have been intriguing epidemiologic leads under the category of clustering. As noted above, time-space clusters of cancer are only likely to be worthy of consideration with rare aggressive tumors which have a short latent period, such as acute leukemia [83], Burkitt’s lymphoma [84, 85], and inflammatory breast cancer [86, 87]. Thus far, investigation of these clusters has rarely, if ever, shown to have a specific cause. An increase in a rare cancer not necessarily in a cluster can be a fruitful finding. For example, investigations of vaginal cancer in young women showed an etiologic role for diethylstilbestrol [121]. Mapping of cancer mortality has been useful, successfully showing asbestos exposure, especially associated with smoking, as the cause of mesothelioma in shipyard workers [122]. Cancer mortality mapping also led to studies showing that tobacco chewing caused oral cancer in women in the southeast United States [123]. For specific viruses, the model for cancer clustering linked to a virus is HTLV-I, which could be linked to adult T-cell leukemia/lymphoma. It is not readily transmissible and therefore has a limited distribution.

The studies of interest in Hodgkin lymphoma and brain cancer are provocative because they indicate groups of patients where studies should be focused in determining a possible viral etiology. For Hodgkin lymphoma, the prominence of clustering of cases reported by Vianna et al. [124, 125] focus on a group that is most probably not EBV-associated: high school students with nodular sclerosing HL. EBV is predominantly associated with Hodgkin lymphoma in childhood and in the elderly and with the histological characterization as mixed cellularity histological subtype. For brain cancer, the increased incidence of brain tumors in northern Nevada [106, 107, 110] following an outbreak of chronic fatigue syndrome previously linked to HHV-6 [71] may represent “clustering”. Attention should be given to identifying specific groups for intensive study rather than all patients with the tumor. This is particularly applicable to oral cancer, the one tumor where it has been suggested that the virus is present in every tumor cell but not normal cells [118]. This study used tumors from an area in India with elevated HHV-6 titers in the healthy population. It could be suggested that attention should be given to patients in areas with elevated HHV-6 titers [63] as possibly being more fruitful than low titer populations.

It should be noted that considerable attention is being given to properties of HHV-6 consistent with oncogenic potential such as oncomodulation and chromosomal integration [67, 119]. These properties are also present in non-oncogenic viruses, however, and therefore at this time we believe a focus on mechanisms of HHV-6 oncogenicity are premature.

It is important to consider the viral differences between EBV and HHV-6, one being a gamma herpesvirus and the other a beta herpesvirus, respectively. Each virus has biological and genetic differences, which may translate into different oncologic mechanisms that affect the acceleration or development of cancer. An in-depth description of these differences is beyond the scope of this review.

## Conclusion

There is insufficient evidence to indicate HHV-6 is an etiologic agent with respect to Hodgkin lymphoma and brain cancers. Based on minor criteria, HHV-6 may play some role in the development of these two malignancies. Studies report evidence for both HHV-6A and HHV-6B being involved in HL [77–79] and brain cancer [89, 90, 92]. Therefore, we suggest classifying both HHV-6A and HHV-6B as IARC Group 2B, possibly oncogenic for humans.

We suggest that methods demonstrating EBV oncogenicity be applied to HHV-6. In addition, there is at least one other cancer, oral cancer, where one of our major criteria has reportedly been fulfilled. Therefore HHV-6 can still be considered as a possible human oncogenic virus.

Although the evidence for HHV-6 as an etiologic agent for Hodgkin lymphoma and brain cancer is currently weak, there are two important opportunities to strengthen the case for it being designated as a Class I carcinogen by IARC. First, it could be helpful if some of the studies noted as supporting the role of EBV in oncogenesis, such as the development of prospective studies in high-risk populations, are implemented for HHV-6. Second, additional studies of oral cancer in Indian populations with elevated HHV-6 antibodies could confirm the importance of the study indicating the presence of HHV-6 in all of the tumor cells [118].

## Data Availability

Not applicable

## Abbreviations

ATLL: Adult T-cell leukemia/lymphoma
BL: Burkitt lymphoma
EBV: Epstein-Barr virus
HAM: HTLV-I associated myelopathy
HBV: Hepatitis B virus
HBV: Hepatitis C virus
HHV-6: Human herpesvirus-6
HHV-7: Human herpesvirus 7
HHV-8: Human herpesvirus 8
HIV: Human immunodeficiency virus
HL: Hodgkin lymphoma
HPV: Human papilloma virus
HSV-2: Herpes simplex virus 2
HTLV- I: Human T-cell lymphotropic Virus Type-I
IARC: International Agency for Research on Cancer
KSHV: Kaposi’s sarcoma herpesvirus
MCV: Merkel cell tumor virus
NPC: Nasopharyngeal carcinoma
PCR: Polymerase chain reaction
RS: Reed-sternberg
SHML: Sinus histiocytosis with massive lymphadenopathy
